# Cold Plasma-Enabled Interface Engineering and In-Situ Functionalization of Printable Feedstocks in Additive Manufacturing: Mechanisms, Materials, and Applications

**DOI:** 10.3390/pharmaceutics18070870

**Published:** 2026-07-16

**Authors:** Xhoi Xibri, Giuseppe F. Racaniello, Brendan Gilmore, Nunzio Denora, Dimitrios A. Lamprou

**Affiliations:** 1 School of Pharmacy, Queen’s University Belfast, Belfast BT9 7BL, UK; xhoi.xibri@uniba.it (X.X.); b.gilmore@qub.ac.uk (B.G.); 2 Department of Pharmacy-Pharmaceutical Sciences, University of Bari Aldo Moro, Orabona Street 4, 70125 Bari, Italy; giuseppe.racaniello@uniba.it

**Keywords:** cold plasma, additive manufacturing, surface functionalization, interface engineering, drug delivery systems, process validation, Quality by Design, Critical Quality Attributes, Critical Process Attributes

## Abstract

Cold plasma (CP) has emerged as a multifunctional surface engineering technology capable of enabling precise, non-thermal modification of material interfaces, while additive manufacturing (AM) has transformed modern fabrication through a layer-by-layer model of personalized therapies. This review discusses potential interactions between cold plasma technologies and additive manufacturing processes for pharmaceutical and biomedical applications. CP has been investigated for surface modifications before and after manufacturing processes in several material science applications. Through controlled surface activation and plasma-induced chemistry, CP-assisted processes can enhance interlayer adhesion, surface wettability, antimicrobial activity, and bioactivity of AM-fabricated models, by generating reactive species and introducing functional groups into the surfaces of materials. The review also discusses engineering and regulatory challenges associated with plasma technologies in AM. Overall, CP represents a versatile surface modification technology whose interaction with materials used in AM deserves further investigation.

## 1. Introduction

Alongside emerging technologies which have attracted significant attention for their applications in precision medicine and targeted therapies, plasma-based techniques have also emerged as a very flexible and impactful approach. The unique physicochemical properties of plasma enable precise control over surface modifications, antimicrobial control, and chemical reactions, positioning plasma technology as a critical tool for advancing both principal research and applied sciences. Plasma is the fourth state of matter. It is a fractionally ionized gas composed of electrons, ions, and neutral particles [1]. Langmuir was the first to use the term “plasma” for this ionized gas, in 1927. The two primary categories of plasma classification are non-thermal plasma (also known as low-temperature or cold plasma) and thermal plasma (high-temperature or hot plasma). Plasma treatment is utilized to modify the surface properties of various materials; consequently, it finds applications in diverse fields, including pharmacology, medicine, and tissue engineering. The most important advantage of this method is its ability to modify the surface of a polymer without altering the bulk properties of the material, thereby preserving the original mechanical characteristics [2].

Particularly, cold plasma (CP) is a technology generated by exposing a low-pressure gas to an electromagnetic field at radio or microwave frequencies, producing plasma at low temperatures [3]. This type of plasma consists of excited molecules, ions, free radicals, and ultraviolet (UV) photons, generated through high-energy electron interactions, within a gas-filled chamber under atmospheric or low pressure. CP is extensively employed in medical fields such as blood coagulation, wound healing, microbial reduction, and tumor therapy, due to its cost-effectiveness and operational simplicity [4]. Its surface engineering ability can be applied to numerous polymers, improving their physical and chemical characteristics for drug delivery, mucoadhesion, and antimicrobial activity. Consequently, CP technology offers a versatile and efficient approach for modifying a wide range of heat-sensitive and nonwoven medical and biomedical materials, advancing pharmaceutical and biomedical progress. These properties support the use of CP as a versatile surface modification tool in several areas of pharmaceutical and biomedical research. Considering individual differences in drug response, AM has been demonstrated to enhance therapeutic efficacy, reduce adverse effects, and improve patient adherence and treatment outcomes [5]. While AM has become a widely adopted fabrication technology, some limitations remain unaddressed. In the context of pharmaceutical and biomedical materials, CP has been investigated mainly as a surface treatment to modify wettability, surface chemistry, antimicrobial behavior, and biological interactions. However, these benefits must be assessed considering potential risks, including active pharmaceutical ingredient (API) oxidation, formulation alterations, solid-state changes, residual reactive species, and batch variability due to the sensitivity of plasma parameters. Existing strategies to improve interfacial bonding and surface functionality are limited in AM (Table 4). Physical approaches, including laser-assisted localized heating and infrared (IR)-preheating, enhance interlayer adhesion with high temperatures and melting points, but they may induce polymer degradation, dimensional deformity, thermal variations, or loss of bioactivity, particularly for heat-sensitive pharmaceuticals and biomaterials. Chemical strategies, like primers and additives, improve adhesion and rheological behavior, yet they introduce additional excipients; thus, the core formulations can be modified, which may induce cytotoxicity, instability of APIs, and lack of reproducibility. Most reported applications involve CP treatment of material surfaces or final printed constructs, mainly as pre- or post-fabrication surface modification strategies. CP offers a unique and different approach, through non-thermal operation, by running at low temperatures. It provides spatially selective treatment in micro- or nanoscale depth and enables “chemistry-on-demand” through controlled generation of reactive oxygen and nitrogen species, allowing functional group incorporation and a surface activation as a solvent-free method. Its integration remains limited due to challenges related to synchronization of plasma chemistry with printed matrices, lack of standardized treatment protocols, and insufficient understanding of CP’s effects on pharmaceutical formulations during layer-by-layer (LbL) fabrication.

**Figure 1:**
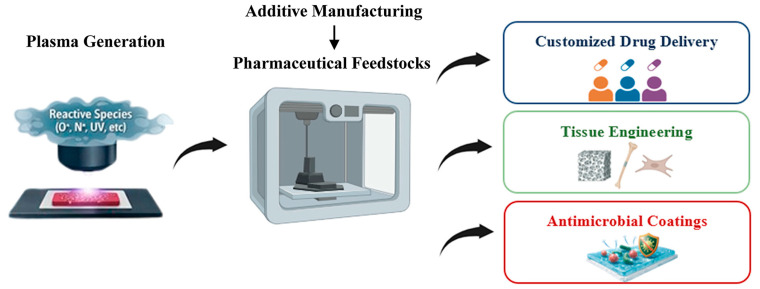
Conceptual schematic of plasma-assisted AM for pharmaceutical and biomedical applications.

This review goes beyond the current knowledge by showing CP not only as a surface treatment used before or after printing, but also as a tool that can be integrated directly into the printing process. At the same time, it extends AM beyond geometry-based fabrication by introducing controlled surface chemistry, interfacial engineering, antimicrobial activity, and biofunctional modulation as part of the manufacturing process. This integrated CP-AM approach therefore represents a transition from passive LbL fabrication toward active, digitally controlled, and AI-assisted manufacturing of pharmaceutical and biomedical constructs. Figure 1 represents the principal outcomes of CP treatment applied to AM products in pharmaceutical and healthcare sectors.

## 2. Methods

A structured and comprehensive literature survey was conducted to support the main framework and organization of this review. Review and article publications were obtained from major scientific databases, including PubMed, Advanced Therapeutics, Nature Biotechnology, ScienceDirect, Scopus, Web of Science Core Collection, and IEEE Xplore, as data sources, with confirmation of citations and recent publications via Google Scholar. The research method combined specific vocabulary and keywords related to cold plasma technologies (“cold plasma”, “non-thermal plasma”, “low-pressure plasma”, “plasma surface modification”, “plasma polymerization”, “plasma sterilization”) with additive manufacturing descriptors (“additive manufacturing”, “3D printing”, “bioprinting”, “material extrusion”, “fused deposition modelling”) and application-related terms (“drug delivery”, “bioink”, “surface functionalization”, “interlayer adhesion”, “interface engineering”, “sterilization”, “tissue engineering”). The considered timeframe for publications covered a wide interval (from 2000 to 2025, with the highest content from 2020–2023), in order to track the evolution of both technologies, while earlier works were included to provide historical and mechanistic context and fundamental well-established principles. Only completed articles published in English were considered.

## 3. Fundamentals of Cold Plasma Technology

As a fully developed surface engineering technology, CP operates through well- characterized physicochemical mechanisms that govern its interactions with different materials. Diverse plasma generation devices provide controlled operational parameters that define plasma composition and overall treatment performance.

### 3.1. Principles, Attributes, and Mechanisms

CP is a non-thermal, highly reactive technology that enables surface modification through electron-driven chemistry rather than heat-based processing. Its principal mechanism is based on the generation of reactive oxygen and nitrogen species (ROS/RNS) and electric/electromagnetic charged particles, as well as UV photons, which trigger surface-related modifications, including oxidation, surface etching, crosslinking, radical formation, thin-film deposition, and grafting [6]. CP has been widely applied in polymer surface modification, optimization, antimicrobial activity, and regulation of drug release, as well as wound treatment, blood coagulation, and emerging anticancer therapies [7], as well as in environmental and pharmaceutical contexts, including the degradation of pharmaceutical contaminants such as sulfonamides [8] and β-lactam antibiotics [9], as well as other active compounds, in wastewater systems [8,10].

From a materials science perspective, CP primarily modulates drug release behavior and material performance through surface-driven mechanisms. Surface modifications induced by CP may influence wettability, surface energy, and other interface-related properties depending on the material and the processing conditions. Compared with several conventional surface modification strategies, CP can provide solvent-free, surface-selective treatment under relatively mild thermal conditions. However, its effects remain strongly dependent on the treated material, plasma source, and operating parameters. CP is not always a superior method when compared to other conventional approaches, as its surface-limited properties may limit its application in cases where sterilization for whole printed products or high interlayer bonding is required.

### 3.2. Generation Methods and Devices

CP parameters are determined by electrode configuration, power supply, operating pressure, and gas composition. The generation methods are broadly classified into electrode-based discharges and electromagnetic field-driven systems [11]. Dielectric barrier discharges (DBDs) and corona discharges are widely used due to their simplicity, scalability, and suitability for large-area treatments, while (Atmospheric Pressure Plasma Jet) APPJs enable localized surface modification. Recent developments, including micro-plasma arrays [12] and on-chip plasma sources [13,14], are designed for precise, localized treatments and integration into more advanced AM systems. The selection of the plasma generation method depends on the intended application, including treatment area, plasma uniformity, gas chemistry, and thermal limitations. A thorough understanding of device configurations and operating principles is essential for tailoring plasma properties and ensuring efficient performance in biomedical, pharmaceutical, and surface engineering applications. Table 1 explains in more detail plasma sources, their operating conditions, and characteristics for every system.

### 3.3. Cold Plasma–Material Interaction Mechanisms

The overall plasma–material interaction is governed by the combination of discharge parameters, plasma chemistry, and the intrinsic physicochemical properties of the substrate, enabling controlled surface activation and tailoring without affecting bulk properties. At the microscopic level, these processes occur from electron-impact reactions, ion-energy transfer, and radical-driven surface chemistry. These interactions can generally be classified into physical, chemical, physicochemical, and biomaterial-related mechanisms, leading to ion bombardment and physical sputtering [15], surface radical formation [6,16], functional group incorporation [17,18], plasma-induced crosslinking [19], and plasma polymerization [20,21] or thin-film deposition. From a plasma physics perspective, these effects are strongly governed by the electron energy distribution function (EEDF) [22] and the electron dissociation and ionization levels that control the generation of ROS/RNS. Treatment power and reactive species flux influence the surface energy state.

Despite the demonstrated potential of CP treatments for modifying material surfaces, their translation into pharmaceutical and biomedical applications requires careful consideration of challenges and limitations, including plasma-induced surface aging, hydrophobic recovery, dose-dependent degradation effects, and the material- and device-specific variability that governs treatment outcomes. For instance, in oxygen-atom treatments of polyethylene terephthalate (PET), wettability can decrease by increasing the density of energy applied and then stabilize at ~15° water contact angle (WCA) around an O-atom density of ~1025 m^−2^, beyond which more exposure induces ion-assisted etching and chain scission rather than further functionalization [23]. This behavior demonstrates the kinetics between surface functionalization, plasma-induced ablation, and the plasma dose limits that are often narrow and material-dependent. However, plasma-induced activation is often temporary, due to time-dependent surface aging; in CP-treated materials it is often called hydrophobic recovery. In this process, surfaces that initially become more hydrophilic gradually return toward their original state as polar groups move back into the original material and the surface absorbs contaminants from the surrounding environment [24]. During air-plasma treatment of polypropylene (PP), its hydrophilic nature switched from super-hydrophobic (WCA~152°) to super-hydrophilic (WCA~0°), and it was reported as a stable state for at least 1 week, under atmospheric conditions [25], highlighting that stability depends on power dose and surface morphology. The same aging effect explains the balance between activation and degradation: increasing plasma dose can delay surface recovery by the formation of crosslinked surface layers, whereas excessive ion bombardment can accelerate degradation, leading to brittleness, material loss, or weak surface layers. In contrast, some CP-induced modifications, such as etching, crosslinking, and plasma-assisted polymerization, remain largely stable, particularly in materials such as metals, ceramics, and highly crosslinked polymers.

Importantly, the pressure applied can affect CP–material interaction balances. Low-pressure plasma, which typically operates from ~1 to a few 100 Pa tends to be more spatially uniform over atmospheric plasma systems (operating up to ~10^3^ times greater), with more energy lost as gas heating [26]. These considerations are particularly relevant when treating temperature-sensitive polymeric, pharmaceutical, or biomedical materials. Throughout its applications, these interactions are governed by the transfer of energy and ROS/RNS from the plasma bulk to the surface, which influences process efficiency and selectivity. The plasma sheath, formed due to the higher mobility of electrons, establishes an electric field that accelerates ions towards the material, thereby controlling energy by ion flux and the delivery of radicals [27,28].

In conclusion, recent advances in CP technology, including controlled atmospheric-pressure sources, micro-plasma systems [29], and improved diagnostics [30], have enhanced the precision and reproducibility of plasma–material surface interactions. These interactions are governed by complex and highly parameter-dependent mechanisms that remain insufficiently understood, especially in AM systems. The operational window between beneficial surface functionalization and undesirable polymer degradation is narrow. Furthermore, while many studies report improvements in wettability and adhesion, others highlight hydrophobic recovery or material degradation at higher plasma exposures. These conflicting observations highlight the need for systematic dose-response studies, standardized treatment protocols, and deeper mechanistic understanding to establish reliable processing workflows for different material systems.

## 4. Cold Plasma Applications

Although most studies have focused on CP applications in food packaging and textiles, their findings are highly relevant to AM, as they demonstrate the capability for scalable atmospheric-pressure plasma activation of thermoplastic polymers.

### 4.1. Pharmaceutical, Industrial, and Biomedical Applications

In pharmaceutical sciences, CP has been explored for the modification of excipients and dosage form components, by enabling improvements in material performance, stabilization of drug delivery films, localized surface etching, and overall formulation optimization. Based on the reviewed literature, numerous pharmaceutical excipients that are also employed in AM and 3D printing have been successfully modified using CP technology, resulting in significant improvements in their surface, physicochemical, and functional properties. These materials exhibited common outcomes, such as increased surface roughness, oxygen incorporation, higher surface free energy, enhanced wettability, and improved crystallinity, promoting better cell adhesion and biocompatibility for biomedical and drug delivery applications [17,31,32,33]. The improvements achieved through CP technology for each excipient are summarized in Table 2. Furthermore, CP-based strategies provide a versatile platform for engineering antimicrobial materials and sterilization, demonstrating compatibility with a wide range of substrates, including heat-sensitive polymers, scaffolds, and nonwoven medical textiles, such as wound dressings [34] and surgical meshes [35,36]. It achieves sterilization through a combination of non-thermal physical and chemical mechanisms by generating ROS/RNS (inducing oxidative damage to microbial cell membranes, proteins, and DNA, leading to cell death), UV/VUV radiation (charged particle bombardment), and localized electric field. It acts on the material surface, enabling effective superficial microbial inactivation while preserving the original physicochemical and mechanical properties of heat-sensitive polymers, drug-loaded systems, or biomaterials. Recent studies confirmed that cold atmospheric plasma (CAP), a category of CP operating typically at or near atmospheric pressure, has applications in bacterial, viral, and fungal inactivation, wound healing, dentistry, implant surface treatment, and biofilm control [37]. A comparative device study [38] demonstrated that plasma jets, DBD, and air plasma systems generate distinct reactive species profiles and thermal outputs, leading to significant biofilm reduction with device-dependent tissue penetration and tolerability. Overall, CP efficacy and safety in AM-enabled biomedical constructs depend on careful optimization of the plasma source, reactive species composition, exposure time, power density, and substrate sensitivity, ensuring reproducible surface functionalization and antimicrobial performance without compromising the structural or therapeutic functionality of the printed material. A recent review from Mahmoud et al. [39] presents CP mainly as an antimicrobial technology, due to its ability to inactivate bacteria, fungi, viruses, spores, and multidrug-resistant microorganisms through ROS/RNS generation, membrane disruption, oxidative stress, and damage to proteins and DNA. However, it is also discussed as a sterilization or disinfection method when applied to non-living systems, such as surgical implants, hospital surfaces, food products, and water. It highlights that the main parameters that influence antimicrobial and sterilization efficacy include the plasma source, gas type, voltage, power, frequency, exposure time, gas flow rate, treatment distance, reactive species composition, pH, humidity, surface properties, microbial load, bacterial species, Gram-positive/Gram-negative structure, and biofilm formation. In conclusion, cold plasma can be considered both antimicrobial and sterilizing, depending on the application, although sterilization requires stronger validation to confirm complete microbial elimination [39].

### 4.2. Applications Relevant to Additive Manufacturing

CP treatment is particularly well-suited for polymer-based systems, as it enables selective modification of surface chemistry and micro/nano-topography while preserving bulk structural and mechanical properties. As discussed in previous sections, this results in improved wetting behavior [52], interfacial interactions [51,53], and enhanced surface functionality, as well as sterilization. These attributes are particularly important in the field of AM, where the LbL fabrication approach directly depends on optimized physicochemical and mechanical interactions between successive layers to ensure structural integrity of the three-dimensional (3D) structure [5].

Notably, challenges related to material composition, physicochemical behavior, processability, and reproducibility are frequently faced in AM systems. To address these limitations, researchers have proposed surface modification strategies applied either before material processing or after fabrication of the final construct [54,55,56,57]. Several studies, as earlier stated, have investigated pre-processes, like surface modification strategies by incorporating functional groups, to modify the interfacial properties and processing behavior of polymers. Such approaches are generally aimed at modifying surface wettability, surface chemistry, porosity, and biological interactions of the treated materials or final printed constructs [34,55,58].

Additional CP applications as pre-treatment strategies relevant to AM are associated with bioprinting and hydrogel-based bioinks. A critical and important parameter in bioprinting is preservation of sterility, which is typically obtained through physical methods (including dry heat, autoclaving, irradiation and UV light, and filtration) or chemical methods (including ethanol, glutaraldehyde, formaldehyde treatment, or exposure to ethylene oxide, EtO) [59]. CP can be positioned as a versatile and non-thermal alternative for the pre-sterilization of single polymers, powder blends, or cell-enriched constructs, particularly when working with temperature-sensitive biological materials. Thus, hydrogen peroxide CP gas has demonstrated a surface sterilization technique that utilizes ionized gas species to deactivate pyrogens and pathogenic microorganisms [60], due to the formation of highly reactive hydroxyl radicals, which disrupt membrane lipids, degrade DNA, and damage cellular components, leading to microbial inactivation. Furthermore, the combination of peracetic acid (PAA) with hydrogen peroxide CP gas has demonstrated enhanced sporicidal activity and improved overall antimicrobial efficacy [61]. A major advantage of CP sterilization is its operation under mild conditions, typically below 50 °C and at low pressures around 13.3 Pa [62]. In bioprinting, another fundamental condition is related to the hydrophilicity of initial polymeric blend, especially those related to hydrogel-based bioinks. Hydrophilicity is essential for hydrogel-based bioinks, enabling high water uptake and swelling that mimic the native extracellular matrix (ECM); this supports nutrient and oxygen diffusion, cell viability, and appropriate mechanical properties in printed constructs [63,64]. In this context, a pre-treatment method could be necessary, by increasing surface energy and introducing oxygen-enriched functional groups. As demonstrated in gelatine-based hydrogels [44], “indirect” CP treatment can reduce WCA and increase surface free energy, indicating an increase in water affinity. This enhanced hydrophilic character promotes better water interaction, improved swelling behavior, and greater surface functionality, which are essential for bioprinting applications in tissue engineering, biofabrication, and biomaterial interface optimization.

Overall, CP-assisted strategies yield a promising approach to address some limitations in polymer-based AM systems by enabling controlled surface activation, improved wettability, enhanced interlayer adhesion, and non-thermal sterilization of sensitive materials. Recent advances in plasma source design and process control have further supported its integration into AM workflows, as reported in Table 3. However, the literature reports variability in treatment outcomes, mainly regarding the stability of CP modifications and the balance between functionalization and surface degradation at higher plasma doses.

## 5. Overview of Additive Manufacturing Technologies

Understanding the fundamental well-established principles of AM, including digital design workflows, material selection, and LbL deposition processes, is essential for exploring the integration of complementary technologies such as CP-based surface modification within AM materials. Such integration may further enhance material functionality and processing capabilities.

### 5.1. Principles and Extrusion-Based Techniques

AM, often referred to as 3D Printing (3DP) technology, comprises a group of advanced fabrication technologies that create 3D models due to LbL deposition of initial materials/polymers directly from digital models [66]. AM enables precise control over geometry, internal architecture, and material distribution, therefore facilitating the production of advanced and customized models with minimal material waste [67]. Its LbL fabrication approach requires and enables precise control over internal structure, porosity, and surface characteristics, which are essential for bone-implant integration in biomedicine, controlled drug delivery, and biological performance.

**Figure 2:**
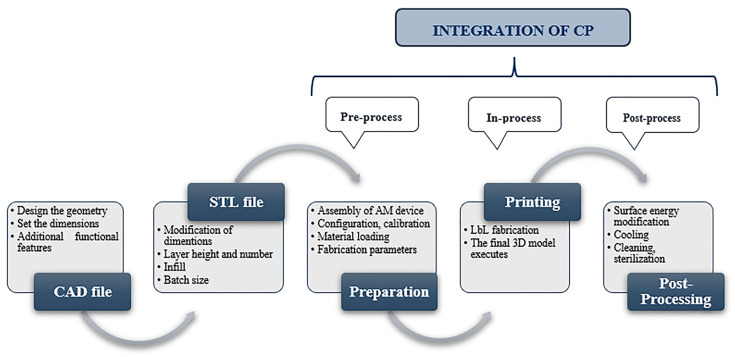
Steps for generating the final product with the integration of CP.

The workflow for generating a digital model prior to fabrication includes some steps common to various applications and manufacturing devices used [68], summarized in Figure 2. It begins with the development of the 3D model with computer-aided design (CAD) software, which allows for the design and definition of the geometry, and finalization with post-processing or the refinement of the LbL-fabricated constructs. Due to the broad spectrum of CP applications, it can be integrated at multiple stages within the fabrication chain of 3D-printed constructs. Specifically, CP has been reported as (i) a pre-treatment method for printable materials and feedstocks, (ii) an in-situ surface treatment applied to deposited layers or to the printed construct during LbL fabrication, and (iii) a post-processing step for surface modification and functionalization of the final printed product.

Pharmaceutical 3DP is increasingly being positioned within both academia and industry for research and practical purposes, due to its low-cost process, short fabrication cycle time, and potential in producing personalized dosage forms tailored to individual patient needs [69]. Its scalability and reproducibility also make it suitable for medium-to-high-volume manufacturing. Current applications include controlled-release tablets, polypills, orodispersible films, gastro-retentive systems, microneedles, and transdermal patches, while bioprinting extends these capabilities to cell-based scaffolds and tissue-engineered platforms for regenerative medicine [5]. In the literature review, there are some principal technologies that are employed in pharmaceutical personalized 3DP, based on the ASTM International standards [69,70], including the following:

1. Material jetting (MJT) or Inkjet printing (thermal and piezoelectric) enables precise deposition of drug solutions/liquids onto substrates.
2. Binder jet printing (drop-on-powder) involves selective deposition of a liquid binder onto a powder bed to form porous tablets.
3. Vat Photopolymerization, including stereolithography (SLA) relies on photopolymerization of liquid resins using UV light to produce precise and complex geometries, but is limited by the small number of pharmaceutically approved photopolymers and the potential toxicity of photo-initiators.
4. Powder Bed Fusion (PBF), including selective laser sintering (SLS), a technique that uses a laser to fuse powder particles into solid structures in a solvent-free, high-resolution process, enabling fabrication of the dosage form.
5. Material Extrusion-based (MEX), the most common and popular technique, involves the extrusion of drug-loaded materials through a nozzle. MEX includes fused deposition modeling (FDM), semi-solid extrusion (SSE), and direct powder extrusion (DPE) techniques.

### 5.2. Bioprinting

With the rapid advancement of 3DP in pharmaceutical sciences, attention has increasingly shifted toward biological and biomedical applications. These require different materials and processing conditions compared with conventional manufacturing, including biocompatible materials such as hydrogels, polymers, bioceramics, and cell-enriched bioinks. Precise control of parameters such as temperature, shear stress, sterility, and structural properties is essential to support cell viability and tissue integration [71]. In this context, CP has emerged as a valuable complementary tool for enhancing 3DP-based biomedical constructs. Basic CP advantages make it a favored tool over conventional modification and sterilization methods in 3D bioprinting. It is compatible with a wide range of biomaterials, particularly heat-sensitive bioink and cell-enriched hydrogels, while enabling surface functionalization under mild treatment conditions.

Ozbolat et al., in their review [72], described bioprinting as a “powerful technology in the fabrication of living tissues and organs for tissue engineering and regenerative medicine, transplantation and clinics, pharmaceutics and high-throughput screening, as well as cancer research”. Unlike conventional 3DP technologies, its focus is on biological–mechanical function, operating under strict physiological conditions. Murphy et al. (2014) [73] proposed three principal 3D bioprinting approaches: (i) biomimicry (aims at replication of native tissue architecture through precise spatial placement of cells and other components), (ii) autonomous self-assembly (cells self-organize into functional tissues, where printed spheroids interact), and (iii) mini-tissue building blocks (uses small and functional tissue units to assemble into larger and complex tissues or organs). Together, these three approaches form the foundational strategies in modern 3D bioprinting and are often combined to achieve clinically relevant tissue constructs.

Generally, bioprinting techniques can be classified into similar categories as other conventional AM. Thus, differing in deposition mechanism, resolution, and bioink requirements, four main categories include [74]:

1. Droplet-based, which generates discrete bioink (typically low-viscosity) droplets through thermal, piezoelectric, or electrostatic actuation, enabling high resolution.
2. Laser-assisted, which is a nozzle-free method in which pulsed laser energy propels cell-encapsulated droplets from a donor substrate to a collector surface; the main disadvantages are high costs and lower scalability.
3. Stereolithography/Digital Light Processing (DLP), which relies on photopolymerization of light-sensitive bioinks, solidifying layers via controlled light exposure, allowing high resolution and structural fidelity.
4. Extrusion-based bioprinting, the most widely used technique, which continuously deposits viscoelastic bioinks through pneumatic, piston, or screw-driven systems, supporting high cell densities and multi-material printing.

The main applications of bioprinting span from the development of cartilage, bone [75], and skin substitutes [76] to more complex constructs such as cardiac patches [77] and vascularized tissues [78,79], designed to support structural repair and functional restoration.

**Table 4:** Current surface modification methods in AM.

Technology	Operating Temperature	Modification Depth	Bulk Property Preservation	Interlayer Adhesion Improvement	Surface Functionalization Control	Compatibility with Heat-Sensitive APIs	Limitations
Cold Plasma (CP)	Low temperatures	Nanometric (10–100 nm)	Excellent	Moderate (in FDM reported 10–40%)	High (-OH, -COOH, -NH_2_)	High	-Limited penetration in depth -Dose-dependent degradation -Hydrophobic recovery
IR-Preheating	High temperatures	Micrometric	Moderate (risk of polymer degradation)	High (tensile strength increased 15–50%)	Low	Low	-Thermal deformation -Risk of API degradation
UV/Ozone Treatment	Low–moderate temperatures	Nanometric	Good	Low–moderate	Moderate	Moderate	-Limited functional groups -Aging effect
Chemical Additives/Grafting	Room temperatures	Micrometric-Bulk	Low–moderate	Moderate	High	Low	-Leaves residues -Cytotoxic
Electron Beam (E-beam)	Moderate–high temperatures	Micrometric-Bulk	Moderate	Moderate	Low–moderate	Low	-High costs -Bulk degradation risks
Thermal Treatment	High temperatures	Bulk levels	Low	Moderate	No	Very low	-API instability and degradation
Solvent-Based Surface Etching	Room temperatures	Micrometric	Low	Moderate	Moderate	Very low	-Toxicity -Not environmental-friendly approach

### 5.3. Challenges and Limitations

Although the field of AM has been established for several decades and has demonstrated broad applicability across different industrial sectors, it remains a technology that needs continuous refinement and optimization, particularly in the context of personalized therapeutic applications in pharmaceutical and biomedical sciences. Current 3DP and bioprinting technologies remain largely focused on geometry-based fabrication, with limited control on surface chemistry and interfacial interactions during LbL deposition. Other challenges that currently limit the expansion of 3DP establishment, in clinical practice, are related to technological and mechanical issues, material-related limitations, device certification, safety concerns, quality controls, process validation, and standardization [69]. The fabrication process begins with selecting suitable materials that meet the desired physicochemical and mechanical properties of the final model. However, many materials are not fully compatible with AM due to factors such as chemical composition, high processing temperatures, or sensitivity to mechanical and physical stresses. Additionally, limitations related to printer architecture, resolution, and material–device interactions can affect print quality, which may lead to structural defects or printing failure, while also increasing material waste, production time, and energy consumption. This issue is particularly critical in polymer-based AM, where recyclability remains limited for many commonly used polymers, thereby increasing environmental and economic impacts [80]. For these reasons, systematic optimization of process parameters, strict standardization of fabrication protocols, and appropriate material or device surface pre-treatments are important steps to take into consideration. Careful control of material quality, environmental conditions, and machine calibration and settings, combined with well-defined validation procedures, can improve reproducibility, structural integrity, and overall manufacturing efficiency. Several limitations are related with 3D bioprinting [81], concerning long-term cell viability and functionality (cell–cell and cell–matrix interactions, tissue maturation) and vascularization (requires blood vessels networks for oxygen and nutrient diffusion). Additional challenges pertain to bioink selection, encompassing printability, biocompatibility, biodegradability, mechanical strength, and structural integrity. Furthermore, standardization and reproducibility remain issues, especially, concerning control printing parameters, cross-linking conditions, and post-processing protocols. Ultimately, scalability and integration with host tissues remain unresolved, largely due to their connection with the immune response, integration, and compatibility, thereby limiting therapeutic applications and clinical translation pathways. In this framework, CP technology may be considered a surface modification strategy for addressing selected surface-related limitations of printable materials. Due to its surface-selective and non-thermal action, CP serves as a surface modification tool for thermal-sensitive materials, bioinks, and cell-enriched products. The modifications potentially lead to improved bonding strength, coating uniformity, antimicrobial and sterilization improvements (critical for bioprinting), and overall structural integrity, as previously mentioned. CP has the potential to enhance reproducibility, reduce failure rates, and support regulatory compliance through a more controlled processing condition. Consequently, applying CP treatments to AM-fabricated materials may help address selected surface-related challenges that currently limit large-scale use and clinical application.

## 6. CP Surface Treatments Applied to Printed Constructs

The integration of CP technology within AM can be extended beyond raw materials and polymer feedstocks pre-treatment, to include both in situ application on printed material surfaces during printing and post-processing modifications of printed products. Together, all these strategies provide flexible and scalable tools to improve print quality, functional performance, and reliability of AM-fabricated products. Table 5 summarizes all outcomes of CP treatment in AM, classified according to the treatment stage.

### 6.1. In Situ Treatments

In situ CP treatments reported in the literature mainly refer to the surface treatment of deposited layers or constructs during fabrication. In these approaches, the plasma source is used to modify the surface of the suitable polymers and materials during the extrusion. In these configurations, CP can be applied to the deposited material during or immediately after layer deposition, thus enabling real-time surface activation, functionalization, or microbial load reduction of the construct under fabrication, offering both functional and manufacturing outcomes, while also requiring additional technical optimization. Compared with post-fabrication treatments, in situ approaches may reduce handling steps and allow surface treatment of layers during construct formation. However, these approaches require careful control of exposure conditions and remain highly material- and device-dependent. This immediate activation is notably favorable for polymer-based systems, where bonding between semi-molten layers affects mechanical integrity. Consequently, improvements in tensile strength and modulus can be achieved. From fabrication perspective, in situ integration of CP reduces handling steps, thus reducing the overall time needed, and contamination risks.

A variety of materials have been employed in techniques that integrate CP in AM, including 3D-printed PLA scaffolds, poly(ethylene glycol) diacrylate (PEGDA) hydrogel scaffolds, and functional inks such as graphene oxide (GO). Owing to the versatility and flexibility of CP devices, a wide range of CP generation sources has been successfully integrated into different AM platforms, demonstrating their technical, manufacturing, and material compatibility. In particular, DBD configurations, combined with jet and corona discharge characteristics, as well as argon-based plasma torches and APPJs, have been effectively incorporated into fused filament fabrication (FFF) and fused deposition modeling (FDM) systems of AM, for the fabrication of surface-functionalized 3D-printed PLA scaffolds [82,83,84]. In the case of nanoparticle-loaded hydrogel systems fabricated via SLA, in situ DBD treatment has been shown to be compatible and effective in enhancing biological performance [85]. Specifically, CP exposure improved human mesenchymal stem cell (hMSC) adhesion and proliferation, and promoted chondrogenic differentiation. Furthermore, an APPJ operating in a DBD configuration has been reported in combination with inkjet printing to treat the deposited material during fabrication [86]. This synchronized approach highlights the potential of CP-AM hybrid systems to achieve fabrication and functionalization within a single and efficient process.

Notably, most of the reported studies focus on microbial load reduction rather than validated sterilization, meeting clinical assurance standards. Low-to-moderate plasma doses may contribute to a reduction in microbial load, whereas higher doses do not necessarily ensure complete sterilization. This dose-dependent nature is partially attributed to the limited penetration depth of CP within 3D-printed constructs. As CP primarily induces surface modifications at the nanometric scale, it is unable to effectively inactivate microorganisms within the internal structure of the material. Increasing the plasma dose to overcome this issue may instead lead to surface degradation. In this context, CP integrated into AM should be regarded as a complementary approach that can support microbial load reduction but cannot fully replace conventional sterilization methods that provide deeper and more reliable sterilization efficacy.

In conclusion, in situ CP treatments reported in the literature should be considered as surface treatment strategies applied to the deposited material without interrupting the manufacturing process. When properly controlled, these approaches may allow localized modulation of surface chemistry, wettability, antimicrobial behavior, and biological interactions of the printed material during LbL fabrication. However, their effects remain highly dependent on the treated substrate, plasma source, gas composition, exposure time, power density, and source-to-surface distance. Careful control of treatment conditions is required to avoid undesired effects such as thermal damage, excessive surface etching, chemical alteration of sensitive biomaterials, loss of bioactivity, or changes in API stability. Moreover, the use of in situ CP treatments requires trained personnel, standardized protocols, reproducible exposure conditions, and appropriate validation before any pharmaceutical or biomedical translation can be considered.

### 6.2. Hybrid Device Engineering

Compared with conventional methods, which include two steps, fabrication and modification, and allow simpler optimization, hybrid CP-AM platforms require more complex device engineering and process validation.

From an engineering perspective, reported configurations generally involve the positioning of a plasma source in proximity to the region where the material has been deposited, allowing the printed layer or construct surface to be exposed under controlled conditions. The architecture of CP devices, including APPJs, DBDs, torches and micro-plasma arrays, makes them suitable for being inserted next to the AM devices [87,88]. Thus, DBD systems can provide more uniform treatment over larger surface areas and are more suitable for coating or modifying planar printed constructs, although their integration may be more challenging for complex geometries due to electrode configuration requirements [89]. Plasma torches generally offer higher reactive species flux, but their potential thermal effects may limit their applicability for thermosensitive pharmaceutical materials and APIs. Micro-plasma arrays, on the other hand, provide miniaturized and spatially controlled plasma deposition, making them promising for high-resolution or localized functionalization; nevertheless, they often require more complex power supply regulation, gas management, and system calibration. All these systems can operate synchronously or successively [90], with controlled spatial and coordinated motion with an AM device. Another engineering strategy involves the development of a single-unit platform equipped with two different functional heads: a printing nozzle and a CP discharge source [91]. In such configuration, product fabrication and CP treatment can occur simultaneously within an LbL deposition model, enabling both interlayer surface modification and outer coating of the final construct. Tablet coating plays a pivotal role in pharmaceutical manufacturing, as it enhances the stability and safety of APIs, masks undesirable taste, modifies drug release profiles, and improves mechanical strength.

Some critical parameters must be considered, including a thorough understanding CP–material interactions, thus determining the operational distance between CP source and the material, gas and flow dynamics, applied power input, and the electric and electronic set-ups of the printer. Particular attention should be given to thermal management, in order to prevent overheating, which can affect material stability, integrity and quality. All the aforementioned specifications and literature examples highlight the need for special attention to be given to hybrid CP-AM engineering systems. In such systems, process parameters must be carefully synchronized and optimized to ensure reliable manufacturing, including the integration of robotic equipment for automated processing [87]. For this instance, micro-plasma deposition in a 3D printing set-up requires modifications in the operating voltage of the power source, the current of the arc applied, the feed speed of planting wires, and the calculated gas consumption, as reported in [87]. Furthermore, validation of the final products should be conducted from the perspectives of both the plasma treatment and the additive manufacturing process.

### 6.3. Post-Processing Treatments

Post-processing with CP is often preferred over pre-treatment to 3D-printed products, as it directly targets the geometry and surface condition of the fabricated final constructs. In the case of pre-treatment, CP is applied to the raw material, prior to printing; thus, subsequent mechanical and physical steps (blending, heating, cross-linking), surface aging effects, and hydrophobic recovery may affect the induced modifications. Conversely, in situ treatment requires highly precise and automated process adjustments to ensure synchronization with material deposition, while post-processing treatment enables CP application directly to the final printed model, allowing greater control over exposure time and dose. This approach minimizes potential interference with printing mechanisms and allows independent optimization. As a result, it facilitates tailored surface modification, controlled porosity and surface roughness, and more reliable sterilization outcomes. It can be considered as an excellent modification strategy toward other conventional post-printing approaches in AM, as it is a dry and reagent-free technology that minimizes chemical waste and reduces contamination risks, supporting sustainability principles. Post-treatment with CP induces highly surface-limited modifications under controlled operations, minimizing undesired outcomes.

In the reviewed literature, CP post-processing treatments have been applied in a wide range of polymer substrates, not only to enhance hydrophilicity by reducing WCA, increasing surface energy and modified surface roughness, but also to overcome some limitations that are faced during the LbL fabrication step in AM. Thereby, CP enables generation of nanoscale surface features that are difficult to achieve during fabrication [92], facilitates the development of antibacterial and anti-biofilm coatings [93], and promotes surface bioactivity [94,95] without altering bulk properties. In the context of tissue engineering and bone regeneration, CP post-treatments have been employed to improve biomineralization capacity, cellular interactions, and overall bioactivity of 3D-printed constructs, thereby enhancing their biological performance and clinical potential [95,96].

The underlying mechanisms of post-processing CP are consistent with those in pre-treatment and in situ approaches, involving the enhancement of surface energy, the incorporation of oxygen-enriched functional groups to improve hydrophilicity and reduce WCA, and the modulation of surface topography and roughness. Importantly, post-processing CP applications has demonstrated additional potential in the enhancement of cell viability for 3D-printed polycaprolactone/eggshell (PCL/ES) scaffolds [96]; improvements in biological incomes, such as increased osteoblasts and mesenchymal stem cells activity in 3D-printed PLA constructs [92]; and improved anti-coagulation and anti-thrombogenic properties on 3D-printed polypropylene (PP) films [94]. To achieve these objectives, a variety of CP sources and device configurations have been demonstrated to be efficient and biocompatible for the abovementioned polymeric systems, including APPJs, CAP and LTP reactors, and argon-based cold atmospheric plasma processes (Ar-CAPP). Each configuration enables precisely controlled surface activation and functionalization, and enhanced bioactivity, as an additional post-fabrication step.

However, as a non-intrinsic modification approach, post-processing CP treatment lacks surface modification depth, affecting only the surface nanometric layers of the final construct, without modifying bulk properties, which may be required in some applications. This becomes more evident in geometrically complex printed models, where the uniform CP penetration into pores and cavities may be difficult. In such cases, limited diffusion of ROS/RNS can lead to non-homogenous surface activation. Furthermore, in biomolecule immobilization and coating formation, there are challenges related to long-term stability and controlled released of grafted agents, particularly under dynamic physiological conditions.

In conclusion, while CP post-treatment is not universally a superior technology, it represents one of the most versatile, controllable, and biocompatible surface modification strategies currently available for tissue engineering and bone regeneration, particularly when surface bioactivity and sterilization must be achieved without compromising structural integrity.

### 6.4. Mechanical Insights

The integration of CP technology into AM-printed constructs can transform the manufacturing approach from a LbL geometric fabrication technique into a material-by-design platform. CP treatment can modify the surface chemistry and topography of printed materials, potentially affecting surface-related mechanical and biological responses. In some cases, CP may reduce the reliance on wet-chemical surface modification methods, thereby improving process sustainability and biocompatibility.

This approach also influences mechanical performance through complex interactions of chemical functionalization, physical surface changes, and the dynamic balance between CP-induced deposition and ablation. Chemically, generated ROS/RNS promote bond scission and radical incorporation on the surface of materials, thus enabling grafting of functional groups, leading to higher surface energy, oxidation degree, or crosslinking. These reactions may influence surface adhesion phenomena and interactions with subsequently applied materials or biological environments. Physically, ion bombardment, UV/VUV photons, and localized electric/electromagnetic fields can induce surface nanoscale roughness or etching, contributing to changes in surface roughness and surface-mediated interactions. However, CP–material interactions are dose-dependent: low- to-moderate power densities typically favor functionalization and thin-film deposition, whereas elevated dose and time exposure may lead to dominant ablation, chain degradation, and loss of plasticity. Therefore, optimizing the deposition–ablation balance is critical to enhance tensile strength and modulus, as well as interfacial bonding, avoiding thermal damage or surface erosion. A controlled CP application protocol ensures the right chemical activation and physical modifications, maintaining mechanical and structural integrity and stability of the AM-fabricated constructs.

## 7. Challenges and Opportunities of CP-Treated AM Feedstocks

The application of CP treatments to AM materials requires standardized protocols and robust process control strategies to ensure reproducible surface modification. The main difficulty lies in synchronizing a highly reactive, dose-sensitive CP process with the temperature-controlled, LbL fabrication process in AM. Precise coordination of CP parameters, such as power density, gas chemistry, exposure time, and nozzle distance with AM dynamics, including temperature, extrusion kinetics, and printing duration, is essential, as the functionalization–degradation range is limited and material-dependent. Hybrid integration also requires redesign of the nozzles, additional gas supply and heat control systems, and the development of control systems that can adjust the CP gas and printing process in real time. Furthermore, the regulatory implications of implementing hybrid CP-AM platforms in pharmaceutical AM and bioprinting applications are highly significant and complex, as these systems merge multiple regulated sectors into a single integrated platform. Firstly, they merge regulations from both pharmaceutical 3DP and plasma-based applications, and in the case of bioprinting, they include regulations governing biomaterials and biological components. Each of these areas is independently subject to strict regulatory oversight, increasing the overall compliance complexity when combined within a single hybrid platform.

### 7.1. Standardization and Process Controls

One of the critical future directions for integrating CP into AM lies in the establishment of standardized protocols and robust process control strategies. At present, variations in plasma parameters, combined with complex AM process settings, may result in inconsistent material responses and limited reproducibility across materials and applications. Systematic adjustments and optimizations of CP parameters adapted to specific materials and AM techniques are necessary to minimize and avoid undesired outcomes, including physicochemical and mechanical alterations. Additionally, it is essential to develop widely accepted treatment methods and platforms, in order to ensure consistent quality control of the process, as well as standardized performance evaluation. Data-driven tools and machine learning offer promising tools for parameter refinement. The integration of real-time monitoring systems can further enhance process stability and control, by enabling closer automatic feedback control during fabrication. Addition advanced tools that can simulate CP–material interactions can also reduce or avoid trial and error during the process. Overall, the establishment of standardized protocols and procedures for CP-integrated AM are fundamental for the transition of this novel hybrid technology from laboratory experimentational scale to industrial production scale.

### 7.2. Regulatory, Device Certification, and GMC/GMP Compatibility

Currently, there are a limited number of CP devices that are certified and commercially available in the market, primarily for therapeutic and dermatological applications rather than integration in AM-printed materials. There are basically produced, manufactured and commercialized in Germany and the UK, as listed below [97]:

1. Argon-driven high-frequency (HF) plasma jet kINPen^®^ MED (neoplas tools GmbH, Greifswald, Germany).
2. Argon-driven microwave plasma torch SteriPlas (ADTEC, Hounslow, UK).
3. PlasmaDerm^®^ (CINOGY GmbH, Duderstadt, Germany), based on a DBD system that uses atmospheric air as the working gas.
4. DBD-based plasma care^®^ (terraplasma medical GmbH, Garching, Germany), also driven in atmospheric air.

**Figure 3:**
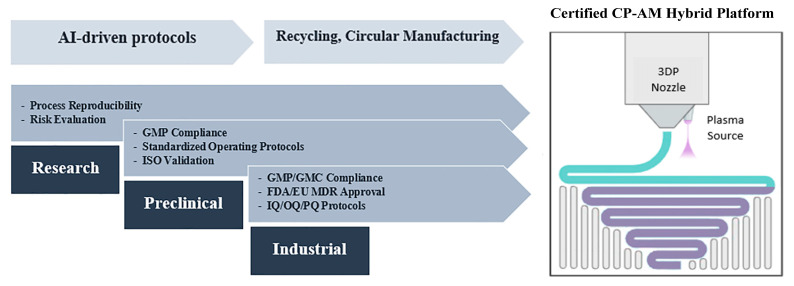
Conceptual roadmap for the development of combination of CP with AM feedstocks.

Additionally, numerous other devices and application methodologies for CP treatments have been patented worldwide [98,99]; however, their certification does not automatically apply to the treatment of pharmaceutical or biomedical printed products. Currently, there is no dedicated regulatory framework for CP-treated printable AM feedstocks, creating unclear classification and validation protocols. However, the regulatory requirements for such platforms should pass through three stages (Figure 3). Firstly, at the research scale, typically operated in academic and research laboratories, regulatory oversight is limited and often not mandatory, but with considerations in process reproducibility, monitoring, and risk evaluation to facilitate further pathways. Secondly, in preclinical development, an integrated CP-AM platform needs to achieve Good Manufacturing Practice (GMP), standardized operating protocols, and ISO validation for biocompatibility; in case of biomedical devices, additional requirements may include ISO 10993-biocompatibility evaluation, as well as ISO 14971-based risk management, addressing potential hazards related with ROS/RNS generation, material degradation, and API stability. Finally, for industrial implementation of a CP-AM hybrid platform, full regulatory compliance becomes mandatory. The systems intended for pharmaceutical manufacturing must comply with GMP requirements under FDA 21 CFR Parts 210/211 [100], while systems for medical devices must follow FDA 21 CFR Part 820 and EU MDR 2017/745 [101]. In bioprinting applications, cell-enriched polymers can be regulated as biologics or as Advanced Therapy Medicinal Products (ATMPs) [102]. Depending on their composition, intended use, and mode of action, CP-treated AM products may in some cases be assessed as combination products, requiring consideration of drug, device, and, where relevant, biologic regulatory requirements. Such an advanced platform must ensure controlled and validated CP parameters, standardized operating set-ups, full GMP/GMC compliance qualification protocols (IQ/OQ/PQ), and complete data recording with full batch traceability [103,104].

Furthermore, while CP demonstrates antimicrobial effects, it needs to be distinguished by validated sterilization, when platforms combining CP and AM devices are intended for pharmaceutical or biomedical applications. The generation of reactive species from CP significantly reduces microbial load. In contrast, sterilization is a regulated and fully validated process that destroys all microorganisms and is defined by sterility assurance levels (SALs) [105]. For this reason, CP integrated in AM can be generally considered as an antimicrobial or decontamination approach rather than validated sterilization, meeting the basic regulatory frameworks, including ISO 14971-based risk management for reactive species exposure, material degradation, and API stability, as well as ISO 10993-biocompatibility testing for CP-modified surfaces [106,107].

The integration of digital control systems also requires compliance with computerized system validation, data integrity, and cybersecurity standards. Therefore, while a CP-AM platform enhances manufacturing capabilities, it increases regulatory complexity, validation requirements, and overall translational costs. In the long-term, it is essential the development of dedicated regulatory guidelines, standardized methodologies, and smart control systems to support a certified CP-AM platform.

### 7.3. Quality by Design and Critical Quality Attributes

The implementation and translation of a system combining CP and AM devices, aimed at pharmaceutical products, especially in personalized therapies, should be guided by Quality by Design (QbD) principles and regulatory frameworks. Rather than end-product evaluation, QbD is a systematic development strategy, in which product quality is “built” in parallel with the design of formulation and manufacturing processes [108]. In the frame of the QbD CP-AM hybrid platform, CP integration into the LbL model offers complex interactions between materials, process parameters, and surface chemistry, during in situ fabrication. These interactions must be systematically identified, monitored, and controlled by Critical Quality Attributes (CQAs), ensuring consistent product performance and manufacturing reliability. CQA principles encompass product attributes for safety, efficacy, and quality, including physical properties (surface morphology, tablet hardness and friability, porosity), chemical properties (drug stability, degradation of APIs, residual solvents), and biological properties (microbial loads, sterility, cytotoxicity, cell viability and adhesion), as well as performance attributes (drug release profile, content uniformity, mechanical integrity) [109]. In addition, in a CP-AM platform, CQAs involve surface energy, wettability, coating adhesion, and microbial load. These attributes must be systematically aligned with Critical Process Parameters (CPPs) that CP technology offers, including plasma power, exposure time, gas composition, and reactive species generation. A thorough understanding of basic mechanisms is essential to monitor surface activation in order minimize risks, like API degradation, design deformity, and undesired modifications and outcomes of the 3D-printed pharmaceutical products. For this reason, Table 6 presents the CQA-based correlations associated with CP treatment outcomes, together with an overview of the key risks and control strategies that should be addressed.

### 7.4. Stability of APIs and Formulations

To ensure the quality, safety, and operational reliability of the hybrid CP-AM platform, developed under QbD principles, additional analytical evaluation is required to be conducted in order to assess the formulation integrity, and, most importantly, the API stability, in 3D-printed pharmaceuticals products. Although the current literature remains limited in this context, potential risks associated with CP treatment of AM feedstocks and AM parameters must be monitored and addressed. In particular, the generation of reactive species or other transient molecules must be carefully conducted when CP parameters are adjusted. Specific chemical interactions may compromise API stability. The safety levels of ROS/RNS can be monitored with relevant chemical markers. Furthermore, despite CP being performed at low temperatures, it may trigger solid-state transformations of the API or excipients that may affect the overall formulation performance. These potential modifications must be controlled with complementary analysis like Differential Scanning Calorimetry (DSC), Fourier Transform Infrared Spectroscopy (FT-IR), X-ray Powder Diffraction (XRPD), and Nuclear Magnetic Resonance Spectroscopy (NMR). A similar finding was reported in several published studies in which zein protein and soy protein isolate were treated with CAP at different voltages and different times, to modify their physicochemical properties, while preserving its functional activity [110,111]. CP treatment in different conditions has different outcomes, especially in oxidation and solid-state transformations, via the mechanisms already discussed in Section 3.3 and Section 6.4. Collectively, the systematic application of these analytical tools is essential to establish robust control strategies and to support the safe and reliable implementation of CP-assisted AM in pharmaceutical production. The existing knowledge gaps in this field may stimulate further research, with future investigations focused to active ingredients rather than being limited to supporting excipients or final manufactured products.

## 8. Future Research Prospects

Future research on CP-treated AM materials should focus on improving treatment reproducibility, understanding plasma-material interactions, and validating safety and performance in pharmaceutical and biomedical applications. To address some of the operational and mechanistic challenges related to precisely integrating CP and AM technologies within a single platform, future developments are highly promising, particularly the rapid advancements in Artificial Intelligence (AI) and its expanding role in advanced technological systems [112]. At present, AI and data-driven modeling support the interpretation of complex CP–material interactions and assist in the optimization of AM treatment parameters for specific substrates and applications, achieving targeted mechanical, chemical, or biological outcomes. In the future CP-AM hybrid platforms, AI could play a more active role through integration of real-time sensors, closer control systems, and predictive computational analytics; it can also strengthen quality assurance and regulatory compliance by detecting anomalies. Such a hybrid approach, in combination with AI strategies, may further support digital synchronization and smart manufacturing. CP parameters can be linked to printing parameters within a unified control structure, enabling automated adjustment, reproducibility, and compatibility with Industry 4.0 frameworks [113,114]. Although AI offers potential for process optimization and reproducibility, the challenges in data infrastructure, in-line monitoring, and regulatory governance must be addressed before AI-driven CP-AM platforms can achieve reliable industrial implementation.

Importantly, this innovative integration may further support recycling strategies and circular manufacturing principles. Independently, both CP-treated product (single polymers or feedstocks) and AM-fabricated products can easily be subjected to further recycling processes. As previously emphasized, CP treatment is a surface modification strategy and the non-altered bulk structure offers possibilities for conventional mechanical recycling. Consequently, in hybrid CP-AM platforms, circular manufacturing strategies primarily involve thermoplastic feedstocks, reactivation of surface aged polymers, as well as AI-assisted techniques for recycled materials. In pharmaceutical and bioprinting applications, recycling strategies must be conducted within regulatory and quality assurance frameworks. Any reuse or reprocessing of materials and polymers must meet GMC/GMP standards, to ensure product quality and validated procedures, particularly for materials intended for biomedical and implantable applications, where patient safety and contamination control are of particular importance [115].

Accordingly, hybrid CP-AM platforms can also contribute to sustainability. CP treatments are generally dry and solvent-free and may reduce the need for wet chemical modification steps, although sustainability claims should be supported by life-cycle or resource-use assessments. By integrating fabrication and surface modification into one unified system, material logistics, post-processing stages, and energy demands can be reduced. Moreover, hybrid systems can enhance resource efficiency through power modulation and pulsed plasma operation, and the implementation of heat recovery systems. Altogether, these attributes position hybrid the CP-AM platform as a promising technology in terms of sustainable and eco-friendly advanced manufacturing.

## 9. Conclusions

Three strategic roadmaps can be identified for the evolution of such hybrid platforms, starting from (i) functional surface enhancement, including pre-, in-, and post-processing CP treatment of fabricated 3D constructs, followed by (ii) integrated hybrid platforms and engineering hybrid CP-AM systems, and concluding with (iii) AI-assisted, certified, GMC/GMP-compliant and sustainable manufacturing production systems. A hybrid CP-AM platform is regarded as an innovative strategy, because it not only improves one existing step of the manufacturing workflow, but it fundamentally transforms surface chemistry and topography within a single operation or coordinated steps through a digitally controlled process. Although both technologies have independently established their roles in pharmaceutical sciences, their integration into a unique platform offers a transformative tool for future manufacturing strategies, even at the industrial production scale.

Despite the potential of hybrid CP-AM platforms in pharmaceutical and biomedical applications, several critical challenges must be addressed before larger-scale clinical and industrial operation. First, the lack of standardized and harmonized process-control protocols limits reproducibility, as both CP parameters and AM variables are highly sensitive and material-dependent. Second, the regulatory considerations and GMC/GMP compliance remain complex, since CP-AM systems merge multiple regulated fields, including pharmaceutical 3D printing, plasma medical devices, and, in the case of bioprinting, biomaterials and biological components, without dedicated certification strategies for hybrid platforms. Third, surface engineering integration presents major technical challenges, requiring precise synchronization of plasma discharge with material deposition, thermal regulations, controlled reactive species generation, and improved real-time monitoring systems. Finally, material and biomaterial stability issues, such as surface aging, penetration depth of CP limited to the surface, and dose-dependent polymer degradation, need further optimization to balance functionalization with structural integrity.

Addressing these challenges through standardization, AI-assisted process controls, and regulatory affairs will be essential to transform CP-AM platforms from the laboratory research scale into more reliable manufacturing strategies. This transition requires laboratory-scale processes such as material screening, CP-printing parameter optimization, surface characterization, stability assessment, and reproducibility studies; preclinical processes including biocompatibility, cytotoxicity, antimicrobial efficacy, degradation, drug-release, ex vivo and in vivo performance testing; and industrial-scale processes involving equipment scale-up, GMP process validation, batch-to-batch quality control, AI-assisted closed-loop control, risk management, and regulatory documentation.

Hybrid CP-AM platforms contribute beyond 3DP, towards 4D functionality, by integrating time-dependent surface functionalization and interfacial engineering [116]. The unique transformative nature of this innovative platform goes beyond the combination of CP advantages with 3DP materials, because it directly reconfigures production workflow through the introduction of real-time functionalization, thereby reshaping the manufacturing paradigm in pharmaceutical sciences.

## Figures and Tables

**Table 1:** Methods and sources of plasma generation.

Category	Device	Power Source	Operating Conditions	Characteristics
Electron-based Discharges	DBD	AC, RF, pulsed	Atmospheric or low pressure	-Surface treatment -Sterilization -Coating -Scalable
	Corona Discharge	High-voltage DC or AC	Atmospheric pressure	-Simple design -Scalable -Surface activation
	DC Discharge	Direct Current (DC)	Mostly atmospheric	-Plasma ignition between electrodes
	AC Discharge	Alternating current (AC)	Atmospheric or low pressure	-Surface modification
Electromagnetic Field–Driven (Electrodeless)	RF Plasma (electrodeless)	Radiofrequency electromagnetic field	Low pressure or controlled atmosphere	-Stable -Uniform plasma -Molecular gases
	Microwave Discharge	Microwave excitation (industrial frequencies)	Low pressure	-Advanced surface treatment -High reactivity
Plasma Jet Systems	APPJ	RF, AC, pulsed, or microwave	Atmospheric pressure	-Coating -High-reactivity processes
	RF Plasma Torch	RF excitation	Atmospheric pressure	-Coating -High-reactivity processes
	Microwave Plasma Torch	Microwave excitation	Atmospheric pressure	-Advanced material processing
Advanced Systems	Micro-plasma Arrays	Various (RF, AC, pulsed)	Atmospheric or low pressure	-Precision surface engineering
	On-chip Plasma Sources	Microfabricated systems	Controlled environments	-Integrated plasma devices -Lab-on-chip -Advanced manufacturing integration

**Table 2:** CP pre-treatment applications fields, materials, and outcomes.

Application Fields	Materials/Polymers	Treatment Type	Surface Modifications	Outcomes/Improvements	Ref.
1. Food packaging	PLA	Atmospheric CP	-Increased surface roughness and surface energy -Oxygen incorporation	-Improved wettability -Enhanced barrier properties and shelf-life	[31]
	Chitosan films	Atmospheric CP	-Surface activization and improved uniformity	-Improved mechanical and barrier properties	[35]
	PET	DBD	-Gradient roughness formation	Improved adhesion, coating performance	[37,40]
	Starch-based films	CP (various gases)	-Surface etching and oxidation	-Enhanced biodegradability, functionality	[40,41,42,43]
2. Environmental	Wastewater	Non-thermal plasma discharge	-Generation of ROS/RNS; oxidative degradation	-Degradation of sulfonamides, β-lactams -Pollutant removal	[8,10]
3. Pharmaceuticals and Drug Delivery	PLA	CP	-Increased crystallinity -Oxygen functional groups	-Enhanced cell adhesion and biocompatibility	[17,32]
	PLA/HA composites	CP	-Increased crystallinity -Surface activization	-Improved osteoconductivity -Improved biomedical performance	[33]
	Starch	CP (DBD, glow discharge)	-Depolymerization -Reduced amylose, crosslinking, viscosity	-Controlled drug release potential -Improved processability	[15,19,40,41,42,43]
	PEEK	Low-pressure Ar/O_2_ plasma	-Reduced contact angle -Surface oxidation	-Improved shear bond strength -Enhanced biological response	[44,45]
	Xanthan gum	Atmospheric plasma	-Increased porosity and surface area -Reduced interfacial tension	-Improved emulsifying capacity and stabilizing properties -Enhance rheology	[46,47]
	Gelatine hydrogels/films	Indirect CP	-Induced crosslinking and surface free energy -Reduced contact angle	-Improved mucoadhesion -Enhanced swelling behavior	[48,49]
	Chitosan-gelatine	Atmospheric DBD plasma	-Surface activation and crosslinking	-Improved antimicrobial activity and biodegradability and controlled drug release	[32,50]
4. Biomedical implants/devices	CFRP	Atmospheric plasma jet	-Surface decontamination -Oxidation-induced functionalization	-Improved antimicrobial properties and integration	[16,51]
	Polypropylene meshes	Cold oxygen plasma	-Surface activization, enabling drug loading	-Improved antimicrobial properties and integration	[32]
	Wound dressing	CAP	-Generation of ROS/RNS and surface sterilization	-Enhanced antimicrobial and antibiofilm activity	[39]

**Table 3:** CP applications related to AM and bioprinting.

Application Fields	Subjects of Treatment	Treatment Type	Surface Modifications	Outcomes/Improvements	Ref.
AM	Polymer feedstocks	Atmospheric plasma	-Surface activation by functional groups	-Improved interlayer adhesion and bonding strength	[54,57]
	Hydrogels	Indirect CP	-Increased hydrophilicity and surface energy	-Improved swelling behavior, ECM-mimicking behavior, cell compatibility	[44,46]
	Nanocomposites	CP	-Surface cleaning and surface activation	-Improved powder fusion and mechanical performance	[58]
Bioprinting	Hydrogel-based bioinks	CP	-Incorporation of oxygen-enriched functional groups -Enhanced wettability	-Improved nutrient diffusion and cell viability	
	Heat-sensitive polymers	Hydrogen peroxide gas plasma	-Formation of hydroxyl radicals -Oxidative damage to microorganisms	-Sterilization below 50 °C -Preservation of bulk properties	[61]
	Cell-enriched constructs	CP	-Microbial inactivation, without heat damage	-Maintained physicochemical integrity -Preservation of bioactivity	[65]

**Table 5:** Classification of CP modifications in AM.

CP Functional Mechanism	AM Stage	Key Effects in AM	Relevant AM Technologies	Typical CP Sources/Techniques
ROS/RNS generation	Pre-process In-process Post-process	-Increased surface energy and wettability -Enhances interlayer adhesion	-MEX -Binder Jetting	-APPJ -DBD -RF plasma
Surface radical formation	In-process	-Stronger interlayer bonding	-MEX	-RF plasma -Atmospheric plasma jet
CP-induced nano-etching	Pre-process Post-process	-Mechanical interlocking -Improved bonding strength -Enhanced cell adhesion	-FDM -Bioprinting	-RF plasma -Microwave plasma -Low-pressure plasma
Plasma polymerization	Post-process	-Enhanced barrier property -Controlled drug release -Surface functionalization	-Pharmaceutical 3DP -SLA	-RF plasma polymerization -Plasma-enhanced chemical vapor deposition (PECVD)
Crosslinking	Post-process	-Stabilized dosage forms -Improved mucoadhesion -Reduced burst release	-SSE	-RF plasma -Atmospheric plasma jets
Surface cleaning or decontamination	Pre-process Post-process	-Improved powder fusion -Enhanced layer bonding -Reduced delamination	-Polymer-based AM	-Atmospheric plasma jets -DBD
Controlled wettability	Pre-process In-process	-Improved bioink spreading and layer bonding -Enhanced powder flowability	-Bioprinting -Binder Jetting	-Atmospheric plasma jets -DBD
UV/VUV photon emission	Pre-process	-Sterilization below 50 °C	-Bioprinting	-Low-pressure RF -Microwave plasma

**Table 6:** CQA-based correlation of CPP with surface modifications and risk mitigation strategies.

CP Parameter (CPP)	Surface Modification Mechanism	Expected Impact on Pharmaceutical CQAs	Key Risks	Control Strategies
Plasma power density	-Radical formation -Oxidation -Nano-etching -Crosslinking	-Improved interlayer adhesion -Coating uniformity -Controlled drug release profiles	-Excessive etching -Polymer chain scission -Loss of mechanical integrity	-Optimize power window -Monitor mechanical properties and surface roughness
Exposure time	-Functional group incorporation -Surface activation	-Improved mucoadhesion -Coating adhesion -Enhanced interfacial bonding	-Surface degradation -Hydrophobic recovery	-Dose-response optimization window -Time-controlled treatment protocols
Gas composition (O_2_, Ar, N_2_, air)	-Generation of specific ROS/RNS species	-Tailored surface chemistry -Improved drug loading capacity -Antimicrobial activity	-API oxidation -Formation of excessive reactive species	-Selection of inert or controlled gas mixtures -Monitoring ROS/RNS levels
Plasma–material distance	-Control of reactive species flux and ion bombardment	-Uniform surface functionalization -Modification across 3D-printed products	-Non-uniform activation -Localized overheating	-Optimized nozzle-plasma alignment -Process calibration
Operating pressure	-Variation in plasma uniformity and species energy	-Controlled surface activation without affecting bulk polymer properties	-Thermal effects -Non-uniform treatment within geometry	-Selection of appropriate plasma source, depending on AM configuration
Treatment environment	-Secondary radical reactions and oxidation pathways	-Modulation of surface energy and stability of induced functional groups	-Uncontrolled surface aging -Hydrophobic recovery	-Controlled environmental conditions -Storage protocols

## Data Availability

No new datasets were generated or analyzed for this review.
